# A novel approach GRNTSTE to reconstruct gene regulatory interactions applied to a case study for rat pineal rhythm gene

**DOI:** 10.1038/s41598-022-14903-6

**Published:** 2022-06-17

**Authors:** Zhenyu Liu, Jing Gao, Tao Li, Yi Jing, Cheng Xu, Zhengtong Zhu, Dongshi Zuo, Junjie Chen

**Affiliations:** 1 College of Computer and Information Engineering, Inner Mongolia Agricultural University, Hohhot, 010018 China; 2 Inner Mongolia Autonomous Region Big Data Center, Hohhot, 010096 China; 3 Inner Mongolia Autonomous Region Key Laboratory of Big Data Research and Application for Agriculture and Animal Husbandry, Hohhot, 010018 China; 4 College of Life Sciences, Inner Mongolia Agricultural University, Hohhot, 010018 China; 5 Faculty of Science, University of New South Wales, Sydney, 2052 Australia

**Keywords:** Bioinformatics, Gene expression analysis, Genomic analysis, Biological techniques, Systems biology, Biochemical networks, Time series, Genomics, Computational biology and bioinformatics, Data mining, Data processing, Gene regulatory networks, Genome informatics

## Abstract

Accurate inference and prediction of gene regulatory network are very important for understanding dynamic cellular processes. The large-scale time series genomics data are helpful to reveal the molecular dynamics and dynamic biological processes of complex biological systems. Firstly, we collected the time series data of the rat pineal gland tissue in the natural state according to a fixed sampling rate, and performed whole-genome sequencing. The large-scale time-series sequencing data set of rat pineal gland was constructed, which includes 480 time points, the time interval between adjacent time points is 3 min, and the sampling period is 24 h. Then, we proposed a new method of constructing gene expression regulatory network, named the gene regulatory network based on time series data and entropy transfer (GRNTSTE) method. The method is based on transfer entropy and large-scale time-series gene expression data to infer the causal regulatory relationship between genes in a data-driven mode. The comparative experiments prove that GRNTSTE has better performance than dynamical gene network inference with ensemble of trees (dynGENIE3) and SCRIBE, and has similar performance to TENET. Meanwhile, we proved that the performance of GRNTSTE is slightly lower than that of SINCERITIES method and better than other gene regulatory network construction methods in BEELINE framework, which is based on the BEELINE data set. Finally, the rat pineal rhythm gene expression regulatory network was constructed by us based on the GRNTSTE method, which provides an important reference for the study of the pineal rhythm mechanism, and is of great significance to the study of the pineal rhythm mechanism.

## Introduction

With the development of sequencing technology, the cost of gene sequencing is getting lower and lower. It is no longer difficult to obtain a large amount of gene sequencing data according to the experimental design. However, large-scale time series genomic data can better understand and study the principles of biological dynamics and molecular dynamics^1–5^. So far, the mechanism of transcriptional regulation in complex systems is still difficult. The main reason is that experiments to verify protein-DNA interactions and their role in regulation are expensive and difficult to replicate^6,7^. Therefore, the methods based on predictive models instead of biological experiments have become one of the effective methods. For example, the inference method of the gene regulatory network (GRN). The GRN can vividly describe the dynamics and biological physiological state of transcription changes. It plays an important role in understanding the genetic basis of phenotypic traits^8–11^.

In the research of gene interaction, the cluster analysis of the whole gene expression profile is one of the important methods to study the expression relationship between genes. First of all, genes with similar transcriptional responses are put together by clustering algorithm, which can explore the interaction of genes involved in similar cellular processes^12^. For example, the co-expression cluster obtained by this method can provide a rough network representation and the co-expression relationship between genes. But, there are only correlations between genes. The causal regulatory relationship between genes cannot be identified. Therefore, the causal regulatory relationship between genes cannot be constructed.

In the past few years, the main process of constructing gene regulatory network is to capture the genes interaction relationships as a network model. The nodes of the network are genes, and the edges represent the interaction relationships between genes^13–15^. In the gene regulation network, the direct interaction between genes represents the causal regulatory relationship between genes. The definition of the network edge depends on the selected method^16^. For example, the linear correlation model based on estimated mRNA abundance can determine the relationship between genes. This method not only lead to false positive edges, but also lose non-linear interaction relationship. Therefore, these models cannot provide reliable biological conclusions based on gene expression data.

In order to reliably reveal dynamic biological processes, the methods for constructing gene regulatory networks are emerging one after another. For example, the ARACNE and MRNET methods are based on mutual information to capture the nonlinear dynamics of gene regulation^17–19^. The BLARS infers the relationship between genes based on punitive linear regression^20,21^. In addition, the GENIE3 of gene network inference with ensemble of trees (GENIE3)^22^ can infer network relationships based on machine learning method. However, in recent years, some new ideas for constructing gene regulatory networks have been proposed, which are based on time series data to infer the direct gene interaction relationships of gene regulatory networks. For example, as an upgraded version of GENIE3, the dynamical GENIE3 (dynGENIE3)^23^ can provide functions for processing short time series data. Moreover, the SWING correlation method proposed based on the Granger causality framework can infer gene regulatory networks based on short time series data^24^.

In addition, the transfer entropy (TE)^25^ is a method for simultaneously estimating linear and nonlinear interactions. It can construct the causal relationship between variables without any assumptions. Because of its advantages and effectiveness in analyzing nonlinear complex systems, it has been widely used in different fields. The transfer entropy has made great achievements for the construction of causality in various fields, such as the field of science and engineering^26–29^, the field of industry^30^, the field of financial^31–39^, the field of brain science^40–45^, the field of climate^46,47^, etc. In addition, transfer entropy is tried to be applied to gene relationship inference^48,49^. For example, the transfer entropy is used to construct the gene regulation network of eukaryotic Saccharomyces cerevisiae by Juan Camilo Castro^50^. In addition, Junil Kim et al.^51^ reconstructed the single-cell gene regulatory network based on transfer entropy, and revealed key regulatory factors from single-cell transcriptome data, which also verified the effectiveness of transfer entropy in constructing gene regulatory networks.

In summary, the construction of gene regulatory networks based on large-scale time series genetic data has become one of the reliable methods for studying dynamic biological processes. Therefore, we propose a new gene regulation network construction method based on time series and transfer entropy, named GRNTSTE, which uses transfer entropy and a large amount of gene expression time series data. Then, we construct a gene regulation network for rat pineal rhythm genes based on GRNTSTE. The gene regulation network reveals the interaction between rat pineal rhythm genes under natural light conditions, which provides a hypothesis for biological experimental verification.

## Methods

The core of GRNTSTE method is the transfer entropy method. However, the transfer entropy is an index proposed based on information theory to measure the asymmetry between process variables. The information entropy and transfer entropy are described as follows.

### Information entropy

As the father of information theory, C. E. Shannon pointed out in the paper "A Mathematical Theory of Communication" published in 1948 that any information has redundancy, and the size of redundancy is related to the occurrence probability or uncertainty of each symbol (number, letter or word) in the information^52^. He borrowed the concept of thermodynamics, called the average amount of information excluding redundancy as “Information entropy”. The mathematical expression for calculating information entropy is shown in Eq. (1).

1 $$ H_{x} = - \sum\limits_{x \in \chi } {p(x)\log_{2} } p(x) $$

where $p(x)$ denotes probability and $\chi$ contains all the possible realizations of $x$. Information entropy is a quantity used to measure the uncertainty of the system. The more uncertain the system being observed, the greater the information entropy, and the more stable the system, the smaller the information entropy.

### Transfer entropy algorithm

In 2000, S. Thomas proposed the concept of transfer entropy^25^ based on the theory of information entropy. Transfer entropy is often used to describe the transfer of information between process variables, and it can be calculated how much this information transfer can reduce the uncertainty of the observed system. For example, when the transfer entropy from variable $x$ to variable $y$ is greater than the transfer entropy from variable $y$ to variable $x$, then $x$ is called the cause and $y$ is called the effect. Therefore, we can establish a causal driving relationship between two variables according to this rule. However, the application of transfer entropy needs a lot of time series data.

Since the application of transfer entropy requires a relatively large length of time series data, it can only be used in the analysis of neural signals and Electroencephalogram data in an era when the amount of data is generally small. However, with the development of the era of big data, data has gradually become an asset to be possessed. Therefore, various fields have gradually realized the importance of data, and collected and accumulated a large amount of time series data based on reasonable design. We believe that transfer entropy will become one of the important methods to analyze the causal driving relationship of time series data in the future.

The transfer entropy is used to measure the asymmetry between time series variables based on conditional distributions, which produces a causal relationship between drive and response. In addition, the equivalence between transfer entropy and Granger causality test has been proved. The transfer entropy can handle nonlinear time series data well and is very sensitive to Granger causality. Since transfer entropy considers the transfer of information between time series variables without assuming a specific relationship between variables, it has better applicability than Wiener-Granger causality, especially for nonlinear systems. The formula for transferring entropy is shown in Eqs. (2) and (3).

2 $$ \begin{aligned} T_{y \to x} & = \sum\limits_{x,y} {p(x_{n + 1} ,x_{n}^{(k)} ,y_{n}^{(l)} )} {\kern 1pt} {\kern 1pt} \times \log \frac{{p(x_{n + 1} ,x_{n}^{(k)} ,y_{n}^{(l)} )}}{{p(x_{n + 1} |x_{n}^{(k)} )p(x_{n}^{(k)} ,y_{n}^{(l)} )}} \\ & = \sum\limits_{x,y} {p(x_{n + 1} ,x_{n}^{(k)} ,y_{n}^{(l)} )} {\kern 1pt} \times \log \frac{{p(x_{n + 1} ,y_{n}^{(l)} |x_{n}^{(k)} )}}{{p(x_{n + 1} |x_{n}^{(k)} )p(y_{n}^{(l)} |x_{n}^{(k)} )}} \\ \end{aligned} $$

3 $$ \left\{ \begin{gathered} x_{n}^{{{(}k{)}}} = \left[ {x_{n} ,x_{{n{ - 1}}} , \ldots ,x_{{n{ - }k + {1}}} } \right] \hfill \\ {\text{y}}_{n}^{{{(}l{)}}} = \left[ {{\text{y}}_{n} ,{\text{y}}_{{n{ - 1}}} , \ldots ,{\text{y}}_{{n{ - }l + {1}}} } \right] \hfill \\ \end{gathered} \right. $$

where $n$ is the length of the time series $x$ and $y$, $k$ and $l$ are the delay lengths of the variables $x$ and $y$ respectively, $x_{n}^{(k)}$ is the $k$ previous states of $x$, and $y_{n}^{(l)}$ is the $l$ previous states of $y$.

The prerequisite for the application of transfer entropy is that the variables in the time series must satisfy the Markov property. When a random process is given the current state and all past states, the conditional probability distribution of its future state depends only on the current state. Let $k$ = $l$ = 1, the variables $x$ and $y$ are first-order Markov processes, which effectively avoids the calculation of high-dimensional probability density functions. The formula is shown in Eq. (4).

4 $$ \begin{aligned} T_{y \to x} & = [ - \sum {p(x_{n + 1} ,x_{n} ,y_{n} )\log (p(x_{n + 1} |x_{n} ))} ] - [ - \sum {p(x_{n + 1} ,x_{n} ,y_{n} )\log (p(x_{n + 1} |x_{n} ,y_{n} ))} ] \\ & = \sum {p(x_{n + 1} ,x_{n} ,y_{n} )} \log \frac{{p(x_{n + 1} |x_{n} ,y_{n} )}}{{p(x_{n + 1} |x_{n} )}} \\ & = \sum {p(x_{n + 1} ,x_{n} ,y_{n} )} \log \frac{{p(x_{n + 1} ,x_{n} ,y_{n} )p(x_{n} )}}{{p(x_{n + 1} ,x_{n} )p(x_{n} ,y_{n} )}} \\ \end{aligned} $$

where $n$ is the length of the time series $x$ and $y$, $x_{n}^{{}}$ is the state of $x$, and $y_{n}^{{}}$ is the state of $y$. The formula (4) reflects the calculation process of the transfer entropy from y to x when the time delay is 1. Similarly, the calculation formula for the transfer entropy from x to y is shown in Eq. (5).

5 $$ T_{{{\text{x}} \to y}} = \sum {p({\text{y}}_{n + 1} ,x_{n} ,y_{n} )} \times \log \frac{{p(y_{n + 1} ,x_{n} ,y_{n} )p(y_{n} )}}{{p(y_{n + 1} ,y_{n} )p(x_{n} ,y_{n} )}} $$

Therefore, the transfer of information entropy in the network can be defined as $(T_{y \to x} - T_{{{\text{x}} \to y}} )$, when $(T_{y \to x} - T_{{{\text{x}} \to y}} ) > 0$, the information flow direction is from $y$ to $x$, otherwise, the information flow direction is from $x$ to $y$.

Therefore, we can get that the two-way information flow of entropy is asymmetry. According to the asymmetry of the information flow, the driving and response factors of the variables can be determined, so as to construct the causal driving relationship. The core advantage of transfer entropy is the direction. We can infer the direction of the causal driving relationship between variables based on time series data.

### Comparison with existing algorithms

#### Experiment on the datasets of DERAM3 challenge

In order to evaluate the effectiveness and accuracy of the GRNTSTE method, We used the Ecoli simulation data set in the DERAM3 challenge for experimental verification. In addition, in order to avoid the randomness of the experimental results, we randomly selected 3 data sets containing 10 genes and 3 data sets containing 50 genes as the experimental data set. These data sets are time series gene expression data composed of 21 points. Then, we construct the sub-network topology interaction relationship of these 6 data sets, and compare and analyze the performance of the algorithms based on gold standards data.

In the sub-network, we regard the transfer entropy value as a directed side information flow. We set different thresholds to calculate the true positive rate and false positive rate at different thresholds, and then calculate the receiving operating characteristic (ROC) curve and calculate the area under the curve. In this way, we can easily evaluate the specificity of the algorithm through the ROC curve. However, it has been noted that small variations from a value of 1 area under the ROC curve can result in large number of false positives^16^. Therefore, the precision and recall (PR) curve and its corresponding area under the curve are also selected to evaluate the performance of the algorithm. In our experiments, we use both ROC and PR curves as metrics to evaluate algorithm performance.

**Figure 1:**
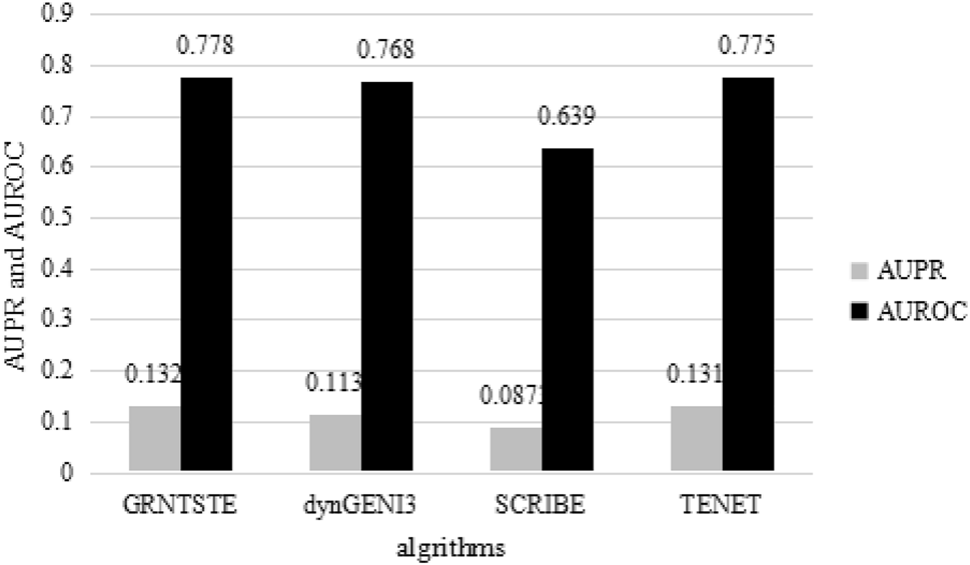
The average value of AUPRC and AUROC obtained for GRNTSTE, dynGENIE3, SCRIBE and TENET algorithms on the datasets with 10 genes.

In order to evaluate the effectiveness of our GRNTSTE method, we compared the GRNTSTE method with the SCRIBE^53^, TENET^50^ and dynGENIE3 algorithms. The SCRIBE, TENET and dynGENIE3 are effective methods to infer gene regulatory networks. For the 6 datasets of the DREAM3 challenge (The DREAM initiative organizes an annual reverse engineering "competition" (we prefer to see it as a community experiment) called the DREAM challenges). The TENET is also a gene regulation network inference method based on transfer entropy. We reconstructed the gene regulatory network based on the SCRIBE, TENET, dynGENIE3 and GRNTSTE methods, respectively. We used the standard convention of calculating the area under the Precision Recall curve (AUPRC) and the area under the receiving operating characteristic (AUROC)^54^. The AUPRC determines the proportion of true positives among all positive predictions (prediction precision) versus the fraction of true positives retrieved among all correct predictions (recall) at varying thresholds. Conversely the AUROC estimates the average true positive rate versus the false positive rate. The Table 1 shows the AUPRC and AUROC values obtained for 3 benchmark networks containing 10 genes. Figure 1 shows the average values of AUPRC and AUROC obtained by the SCRIBE, TENET, dynGENIE3 and GRNTSTE algorithms. It can be seen from Fig. 1 that the AUPRC and AUROC of GRNTSTE algorithm are slightly higher than the SCRIBE and dynGENIE3 algorithms. The GRNTTSTE and TENET algorithm have similar performance.

**Table 1:** Values of AUPRC and AUROC obtained for GRNTSTE, dynGENIE3, SCRIBE and TENET algorithms on the datasets with 10 genes.

Dataset	GRNTSTE (AUPRC)	GRNTSTE (AUROC)	dynGENIE3 (AUPRC)	dynGENIE3 (AUROC)	SCRIBE (AUPRC)	SCRIBE (AUROC)	TENET (AUPRC)	TENET (AUROC)
Dataset 1	0.121	0.783	0.112	0.775	0.091	0.598	0.129	0.743
Dataset 2	0.134	0.756	0.101	0.740	0.078	0.659	0.112	0.798
Dataset 3	0.140	0.796	0.127	0.790	0.093	0.661	0.153	0.785
Average	0.132	0.778	0.113	0.768	0.087	0.639	0.131	0.775

**Figure 2:**
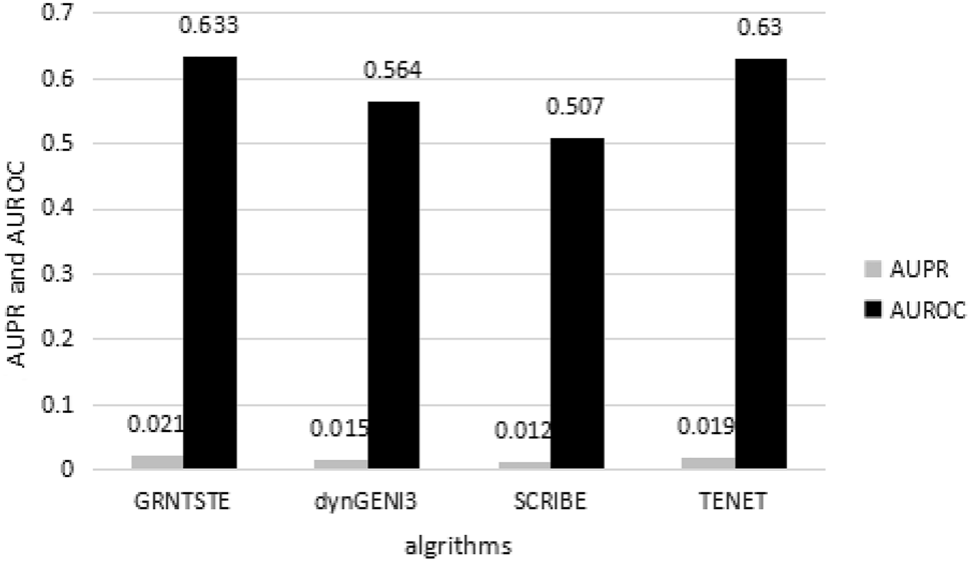
The average value of AUPRC and AUROC obtained for GRNTSTE, dynGENIE3, SCRIBE and TENET algorithms on the datasets with 50 genes.

At the same time, we used dynGENIE3, TENET, SCRIBE and GRNTSTE algorithms to analyze 3 dream challenge datasets containing 50 genes. Table 2 shows the AUPRC and AUROC values obtained by 3 benchmark networks containing 50 genes. Figure 2 shows the average values of AUPRC and AUROC obtained by the dynGENIE3 TENET, SCRIBE and GRNTSTE algorithms. It can be clearly seen from Fig. 2 that the AUPRC and AUROC values of the GRNTSTE algorithm are significantly higher than those of the dynGENIE3 and SCRIBE algorithm. The GRNTTSTE and TENET algorithm have similar performance.

**Table 2:** Values of AUPRC and AUROC obtained for GRNTSTE, dynGENIE3, SCRIBE and TENET algorithms on the datasets with 50 genes.

Dataset	GRNTSTE (AUPR)	GRNTSTE (AUROC)	dynGENIE3 (AUPR)	dynGENIE3 (AUROC)	SCRIBE (AUPR)	SCRIBE (AUROC)	TENET (AUPR)	TENET (AUROC)
Dataset 1	0.021	0.631	0.016	0.588	0.013	0.537	0.020	0.629
Dataset 2	0.021	0.659	0.014	0.547	0.013	0.480	0.018	0.611
Dataset 3	0.021	0.609	0.013	0.556	0.011	0.505	0.019	0.650
Average	0.021	0.633	0.015	0.564	0.012	0.507	0.019	0.630

In summary, we conducted experimental verification based on the DREAM3 challenge open source data set, and the experimental results proved that the performance of the GRNTSTE method is significantly higher than that of the dynGENIE3 and SCRIBE algorithm. In addition, with the increase in the number of genes, the advantages of GRNTSTE are more obvious. However, GRNTSTE and TENET methods have similar performance. Both GRNTSTE and TENET are gene regulation network inference methods based on transfer entropy. It shows the effectiveness and superiority of transfer entropy in the inference of gene regulatory network.

#### Experiment on the datasets of BEELINE

To further verify the performance of the GRNTSTE method, we conducted a comparative analysis of the gene regulatory network construction methods in the GRNTSTE and BEELINE frameworks^55^ and existing effective gene regulation methods. The BEELINE simulation datasets is single-cell gene expression data. The datasets from synthetic networks were created five datasets per parameter set, one each with 100, 200, 500, 2000 and 5000 cells by sampling one cell per simulation. These datasets include 6 different data types, namely LI, linear; CY, cycle; LL, linear long; BF, bifurcating; BFC, bifurcating converging and TF, trifurcating. And we conducted experiments on different algorithms based on the BEELINE datasets, and evaluated the performance of different algorithms based on AUPRC.

**Figure 3:**
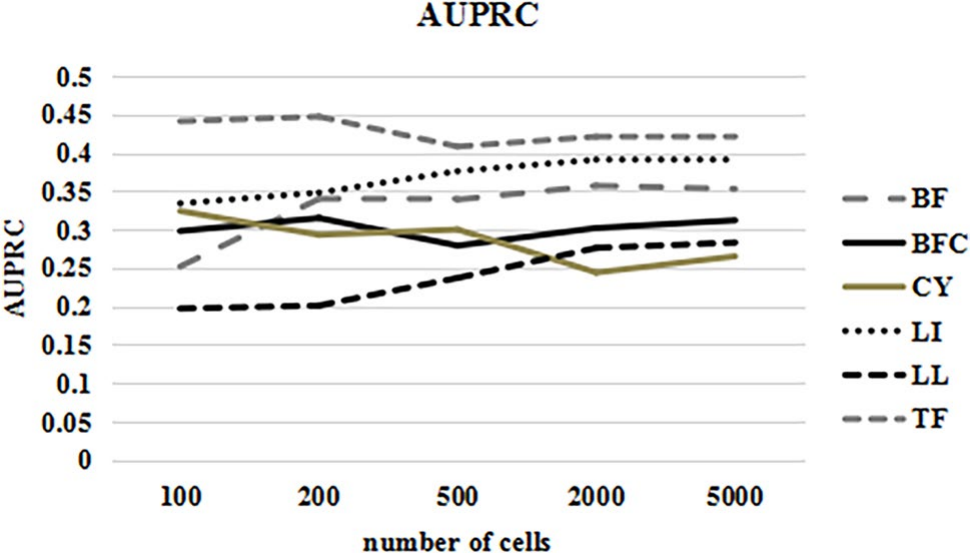
The AUPRC for datasets with different types and containing different numbers of cells.

Since the GRNTSTE method is based on time series datasets to infer gene regulatory networks. We first constructed a pseudo-time-series gene expression dataset based on the simulated dataset and time-lapse information. As shown in Table 3, we then calculated the AUPRC values separately for datasets with different data types and containing different numbers of cells. Figure 3 shows that the AUPRC values obtained by the GRNTSTE method become more stable as the number of cells increases.

**Table 3:** The AUPRC for datasets with different types and containing different numbers of cells.

Number of cells	AUPRC					
	BF	BFC	CY	LI	LL	TF
100	0.252	0.298	0.324	0.334	0.197	0.441
200	0.34	0.315	0.293	0.348	0.201	0.447
500	0.339	0.279	0.3	0.376	0.237	0.408
2000	0.360	0.329	0.348	0.391	0.286	0.455
5000	0.373	0.347	0.369	0.390	0.289	0.456

Furthermore, to compare with existing gene regulatory network construction methods, we calculated AUPRC for datasets of 2000 and 5000 cells, respectively. The detailed results are shown in Table 4. As can be seen from the table, the GRNTSTE method significantly outperforms the SCODE, SCNS, SINGE, GRNBOOST2 and SCRIBE methods, among others. In addition, we further calculated the AUPRC mean of different datasets. It shows that in the BEELINE framework based on the BEELINE dataset, the performance of GRNTSTE is slightly lower than that of the SINCERITIES method, but better than other gene regulatory network construction methods. The experimental results also demonstrate the effectiveness and superiority of GRNTSTE in reconstructing gene regulatory networks based on single-cell gene expression data.

**Table 4:** The AUPRC for BEELINE datasets with different types.

Algorithms	AUPRC						
	LI	CY	LL	BF	BFC	TF	Average
SINCERITIES	0.62	0.51	0.2	0.32	0.33	0.28	**0.377**
GRNTSTE	0.39	0.36	0.29	0.37	0.34	0.46	**0.368**
TENET	0.33	0.38	0.36	0.36	0.30	0.45	0.363
PPCOR	0.38	0.26	0.32	0.36	0.37	0.46	0.358
LEAP	0.56	0.25	0.37	0.23	0.33	0.4	0.357
PIDC	0.4	0.28	0.41	0.26	0.23	0.51	0.348
GENIE3	0.27	0.29	0.36	0.23	0.29	0.46	0.317
SCRIBE	0.37	0.35	0.18	0.38	0.32	0.3	0.317
GRNVBEM	0.51	0.23	0.35	0.22	0.27	0.32	0.317
GRISLI	0.57	0.23	0.14	0.19	0.37	0.34	0.307
GRNBOOST2	0.28	0.31	0.22	0.25	0.31	0.42	0.298
SINGE	0.34	0.36	0.21	0.23	0.18	0.29	0.268
SCNS	0.23	0.26	0.21	0.23	0.22	0.33	0.247
SCODE	0.22	0.31	0.04	0.27	0.19	0.31	0.223

Significant values are in bold.

### Validation on public datasets

**Figure 4:**
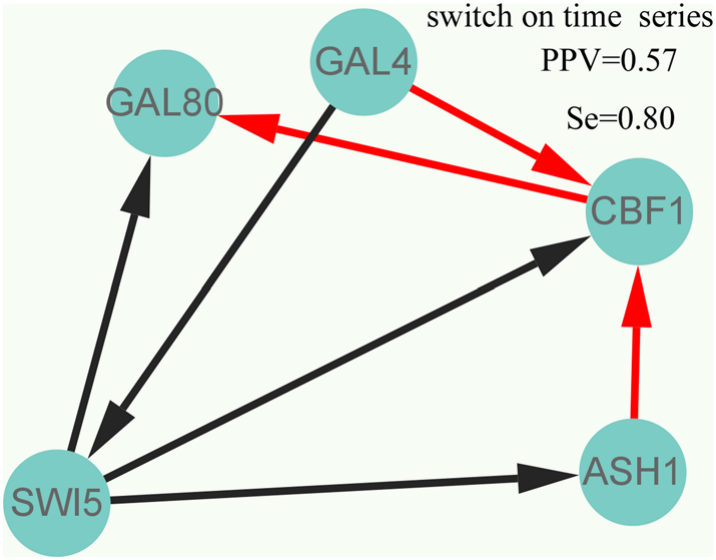
Reverse engineering of the IRMA gene network from time series experimental data using the GRNTSTE approach.

**Figure 5:**
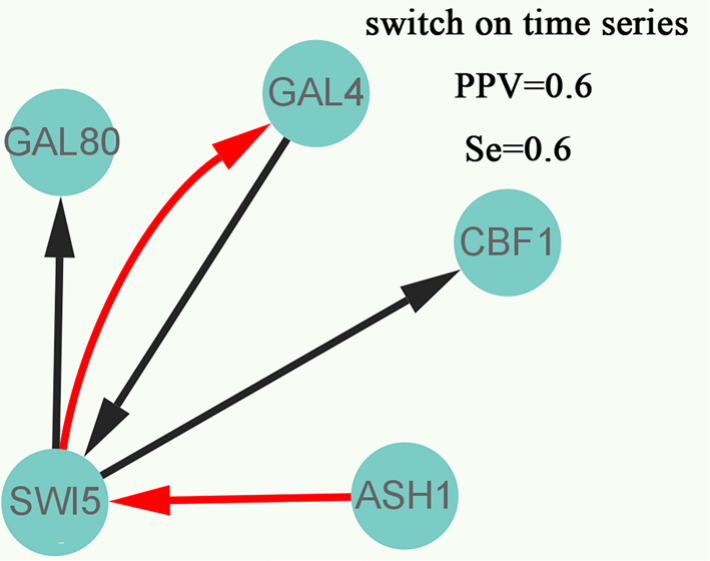
Reverse engineering of the IRMA gene network from time series experimental data using the ODE-based approach.

Furthermore, we further validate the effectiveness of our proposed gene regulatory network inference method GRNTSTE based on public datasets. The datasets named IRMA OFF/ON from Cantone et al.^56^. It includes five genes: SWI5, GAL80, GAL4, CBF1 and ASH1. The method we proposed is to infer the positive regulatory relationship based on time series data, so the IRMA ON data set is selected. Using the gene regulatory network inference method GRNTSTE proposed by us, we constructed the gene regulatory network, as shown in Fig. 4. In addition, we compared GRNTSTE with the ODE-Based Approach proposed by Cantone et al. The result of ODE-Based Approach is shown in Fig. 5. The PPV [Positive Predictive Value = TP/(TP + FP)] and Se [Sensitivity = TP/(TP + FN)] values show the performance of the GRNTSTE and ODE-Based algorithm for an unsigned directed graph. TP, true positive; FN, false negative; FP, false positive. Comparing GRNTSTE and ODE-Based, we found that the GRNTSTE has higher sensitivity when PPV is similar. It shows the effectiveness and superiority of GRNTSTE in the inference of gene regulatory network.

### Ethics approval and consent to participate

All procedures on rat presented in this manuscript were approved by the Institutional Experimental Animal Welfare and Ethics Committee of Inner Mongolia Agricultural University.

## Construction of rhythmic gene regulatory network in rat pineal gland

### Animal model

The study was carried out in compliance with the ARRIVE guidelines (Animal Research: Reporting of In Vivo Experiments). All procedures on rat presented in this manuscript were approved by the Institutional Experimental Animal Welfare and Ethics Committee of Inner Mongolia Agricultural University, based on the method of euthanasia for rat experiments. Then, open the skull, and take out the brain tissues. Next, the pineal gland in the rhythm center was isolated and the second microstructure was identified. Finally, the rat pineal gland was removed and put into a 2 ml Corning freezing tube. At the same time, the details of the sample were marked and immediately put into liquid nitrogen. All rat experiments in our work comply with the national "Experimental Animal Environment and Facilities" standard (GB14925-2010), and follow the "Experimental Animal Management Regulations" (No. 2 Order of the State Science and Technology Commission) and the Ministry of Science and Technology "Experimental Animal License Management Measures" [2001 No. 545]. In our work, we confirm that all our methods are performed in accordance with the above guidelines and regulations. A total of 480 male rats, aged 8 weeks, with an average body-mass index of 180 g, were selected from the rat aquatic breeding farm in Qingdao, Shandong Province. All experimental rats were kept in a 100 square meters independent rat room for two weeks (free feeding, free drinking, and free lighting). In complete circadian rhythm cycle, the rat pineal gland was sampled every three minutes from 7:00 a.m. on November 15, 2020 to 7:00 a.m. on November 16, 2020. It was carried out continuously for 24 h until the end of the experiment. The sampled rat pineal gland was put in a 2 ml Corning Freezer Tube (430,659), cryopreserved in liquid nitrogen immediately. The total RNA was extracted using the Biomend RNApure Rapid RNA Kit (RA103-02). The total RNA extraction results were detected by agilent 2100 with integrity RIN value more than 9.0. A total amount of 3 µg RNA per sample was used as input material for the RNA sample preparations. Sequencing libraries were generated using NEBNext® UltraTM RNA Library Prep Kit (NEB, USA) following manufacturer’s recommendations and index codes were added to attribute sequences to each sample. Briefly, mRNA was purified from total RNA using poly-T oligo-attached magnetic beads. Fragmentation was carried out using divalent cations under elevated temperature in NEBNext First Strand Synthesis Reaction Buffer (5 ×). First strand cDNA was synthesized using random hexamer primer and M-MuLV Reverse Transcriptase (RNase H-). Second strand cDNA synthesis was subsequently performed using DNA polymerase I and RNase H. Remaining overhangs were converted into blunt ends via exonuclease/polymerase activities. After adenylation of 3′ ends of DNA fragments, NEBNext Adaptor with hairpin loop structure were ligated to prepare for hybridization. In order to select cDNA fragments of preferentially 250 ~ 300 bp in length, the library fragments were purified with AMPure XP system (Beckman Coulter, Beverly, USA). Then 3 µl USER Enzyme (NEB, USA) was used with size-selected, adaptor-ligated cDNA at 37 °C for 15 min followed by 5 min at 95 °C before PCR. Then PCR was performed with Phusion High-Fidelity DNA polymerase, Universal PCR primers and Index (X) Primer. At last, PCR products were purified (AMPure XP system) and library quality was assessed on the Agilent Bioanalyzer 2100 system.

### Pineal gland

The pineal gland is the regulatory center of the biological clock. The pineal gland alternately secretes melatonin and serotonin with a distinct circadian rhythm. The pineal gland secretes serotonin during the day and melatonin at night. Since the secretion of melatonin is regulated by light and dark, the light and dark of the circadian cycle also periodically causes changes in melatonin secretion. Studies have shown that plasma melatonin concentrations decrease during the day and increase at night. Therefore, the pineal gland sends time signals to the central nervous system according to the circadian secretion of melatonin, which in turn triggers some time- or age-related biological clock phenomena. For example, sleep and wakefulness in humans, ovulation in the menstrual cycle, and the onset of puberty. Therefore, rhythm genes in the pineal gland play an important role in regulating the rhythmic cycle of organisms.

### The construction of gene regulatory network

**Figure 6:**
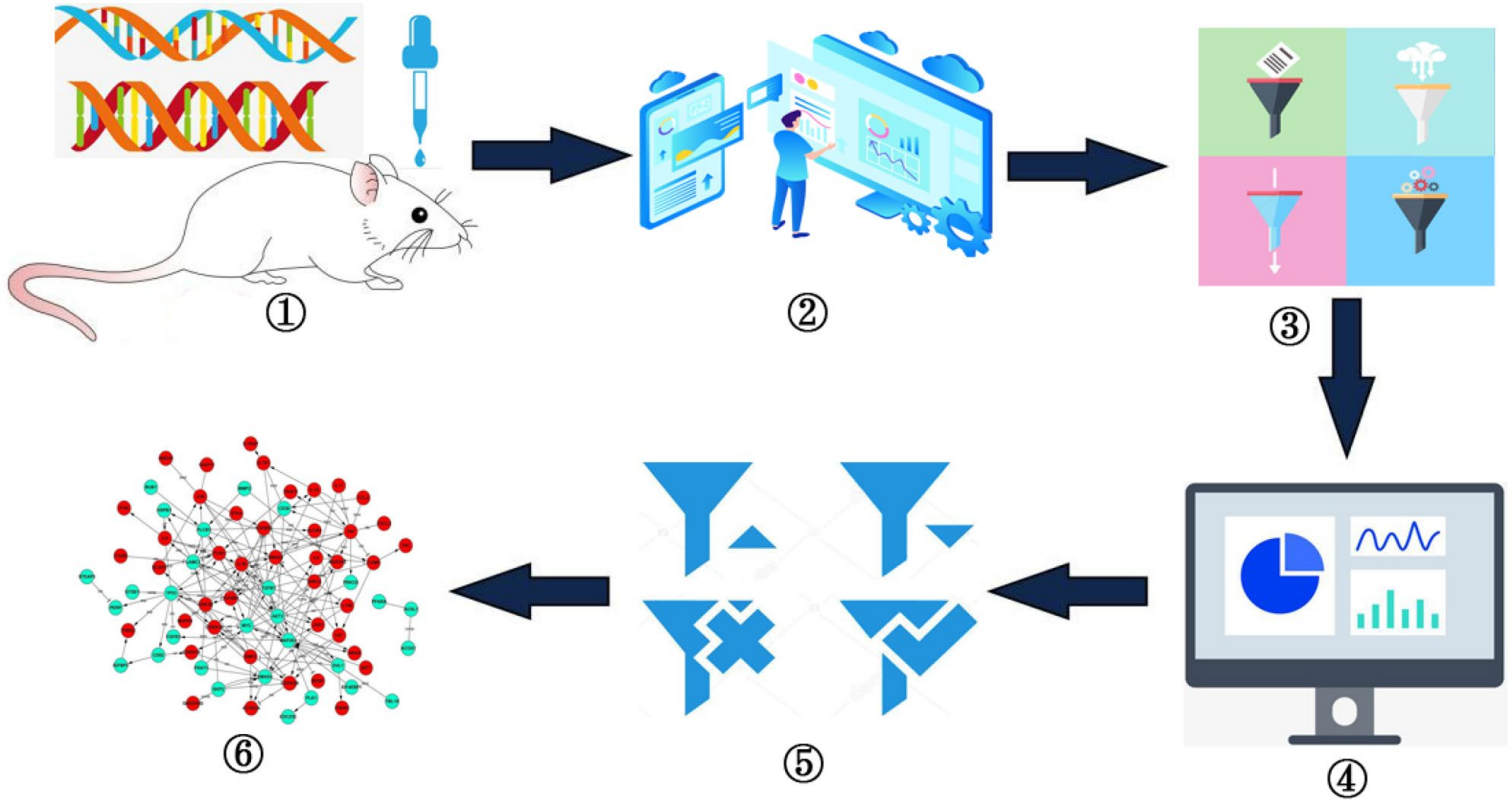
The construction process of gene regulatory network.

In our work, we constructed the rhythm gene regulatory network of rat pineal gland based on GRNTSTE method. And the directed graph is used to describe the regulatory relationship between genes. In this paper, the construction process of gene expression regulatory network is mainly composed of 6 stages. As shown in Fig. 6, it includes the collection of time series data, the selection of target genes, the calculation of transfer entropy, the selection of regulatory relationship and the construction of gene expression regulatory network.

In the stage of sample collection based on time series. In order to understand and study the dynamic biological process of an organism, time series gene expression data can be used to study the state of biological processes at different time points, so as to discover the changing laws of biological processes. Currently, there are two main methods for obtaining time series data in the field of bioinformatics. The first method is single-cell sequencing technology, and the other method is sampling at fixed time intervals.

Single-cell sequencing technology constructs a time-series data set by sampling target tissues and isolating single cells at different growth stages for sequencing. However, there are usually errors in cell separation, and the collected tissue cells cannot construct an equally spaced time series data set, and the cost of single-cell sequencing technology is relatively high. However, there are usually errors in cell separation, and the result data after separation cannot construct an equally spaced time series data set. In addition, the cost of single-cell sequencing technology is very high.

However, the method of sampling by time point according to a fixed sampling rate is a process of sampling the observation target at equal time intervals. Compared with the single-cell sequencing technology, this method has a lower cost, and can obtain sample data at a specified time interval according to the sampling rate of the experimental design, so as to obtain a more accurate and richer sample data set.

For our experimental data collection, we sample the pineal tissue of a rat with the same growth environment, age and sex every 3 min, and the time period is from 7:00 in the morning to 7:00 the next morning. Then, the samples were frozen in liquid nitrogen and sequenced. We collected samples for 24 h, so we obtained 480 rat pineal tissue samples. The n × t (where n is the number of genes and t is the time point) gene expression profile matrix is obtained by genes quantitative analysis.

**Figure 7:**
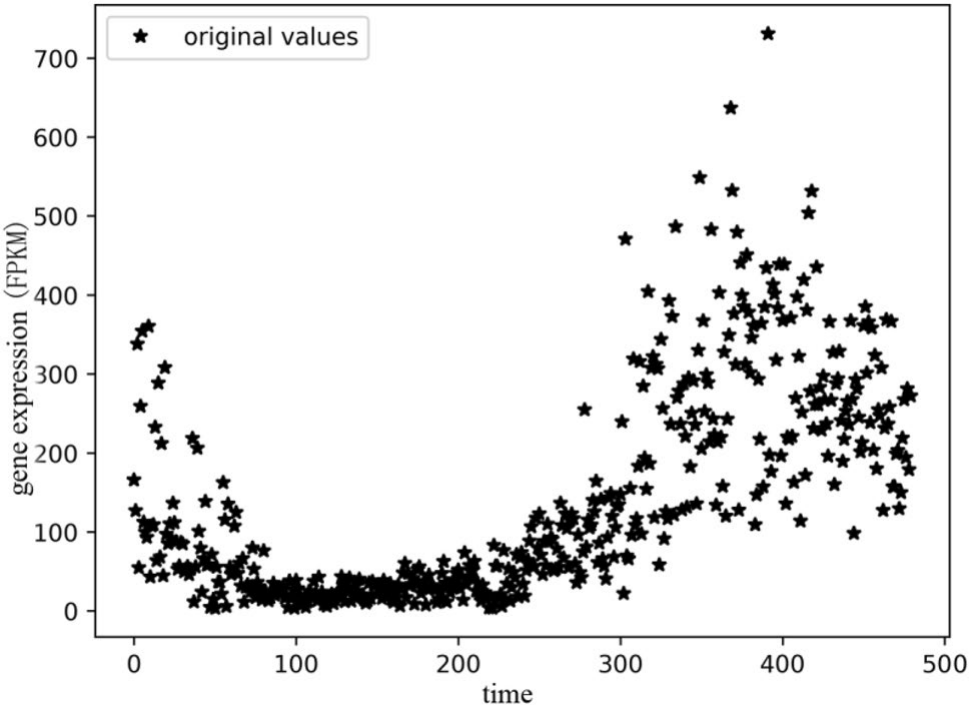
Gene time series expression data, each black star represents the expressed value at the point of time.

In the preprocessing stage of gene time series data, due to the influence of objective environment, equipment and man-made factors in the process of obtaining genetic time series data, there are usually outliers and random values in the data. As shown in Fig. 7, it can be found that there are some random or abnormal values in the gene time series data set.

**Figure 8:**
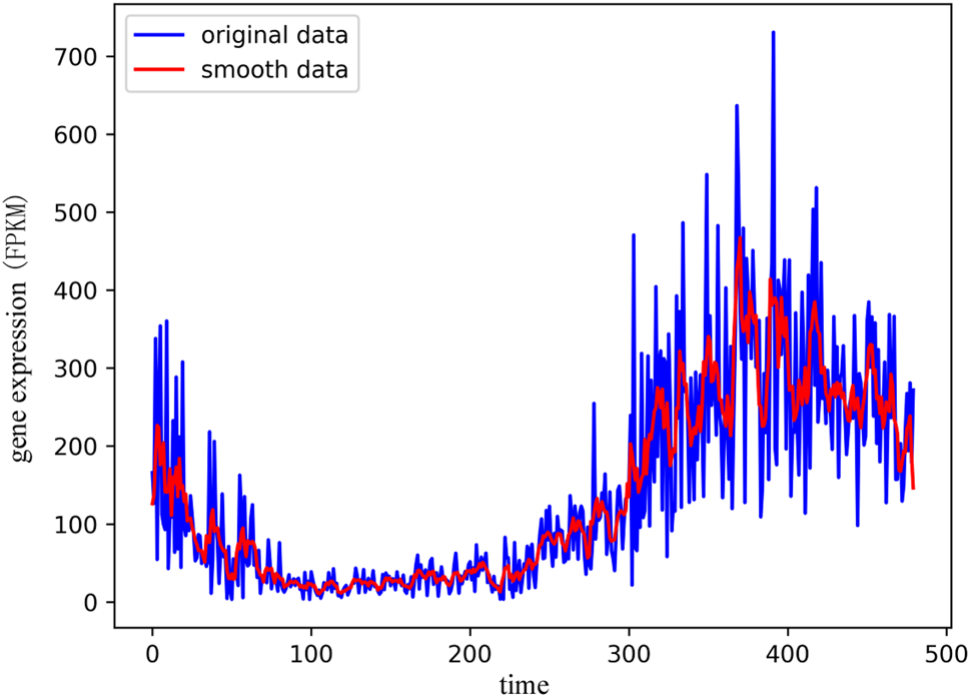
The comparison of gene time series data before and after smoothing, the blue curve represents the distribution of the original time series data, and the red curve represents the data distribution after data smoothing preprocessing.

The existence of outliers or random values will not only affect the accuracy of the calculation results, but also cause the calculation results to deviate from the essential trend of the time series. Therefore, preprocessing is one of the important steps in the data mining process. For the outliers in the time series data, we choose to use the moving average smoothing method to preprocess the collected time series big data, so as to reduce the influence of the outliers on the analysis results. The moving average formula is shown in Eq. (6).

6 $$ F_{t} = (A_{t - n} + ... + A_{t - 1} + A_{t} + A_{t + 1} ... + A_{t - n} )/(2n + 1) $$

where $A_{t}$ represents the actual observed value at time t, $F_{t}$ represents the predicted value at time t, and 2n + 1 represents the size of the smoothing window. We use the moving average smoothing method to smooth the gene time series data, and the smoothing window size is 5, thereby effectively reducing the influence of outliers on the data analysis results. As shown in Fig. 8, it reflects the comparison of gene time series data before and after smoothing. It can be clearly reflected from the figure that the effect of time series data after smoothing is better than before smoothing. Smoothing can effectively reduce the influence of random values on the trend of time series data.

**Figure 9:**
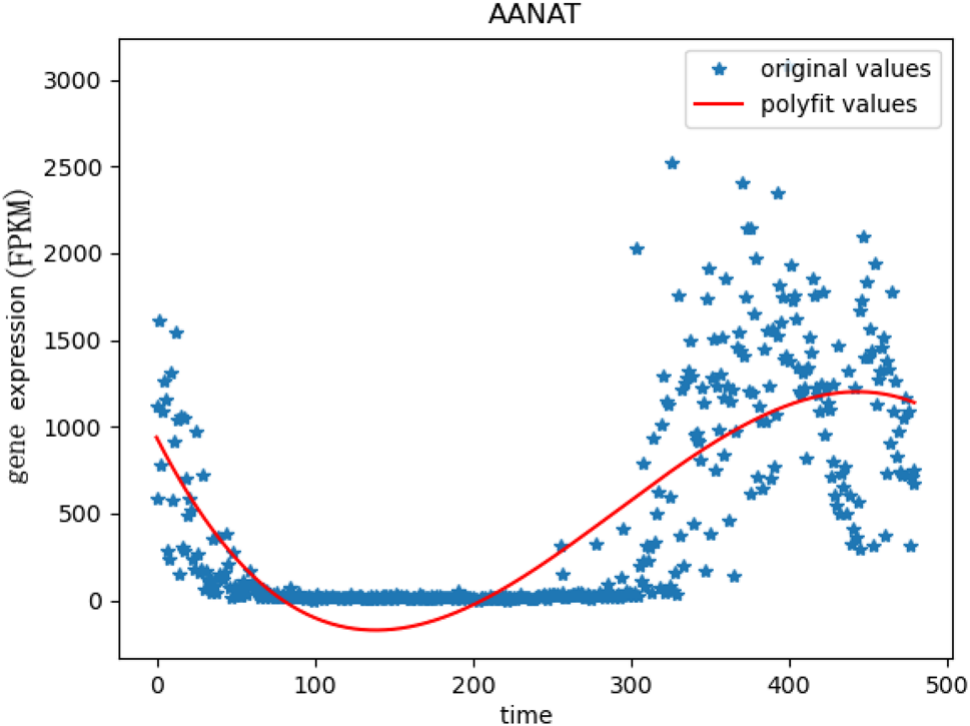
The change trend of AANAT gene expression. The red curve represents the fitting curve, and the stars represent the expression value at the point of time.

In the stage of target gene selection, the target gene selection is an important process in the construction of gene regulatory networks. The target gene we took is the rhythm gene that regulates the secretion of melatonin in the rat pineal gland. In the previous research, the aromatic arylalkylamine N-acetyltransferase(AANAT) gene has been proven to be an important rate-limiting enzyme in the melatonin biosynthesis pathway. The melatonin is an important hormone secreted by pineal gland, and the secretion of melatonin has obvious periodic rhythm. In addition the rhythm of the synthesis of melatonin in the pineal gland is mainly controlled by light, and the change of light is an important sign of the change of day and night. The change trend of AANAT gene expression is shown in Fig. 9. We take the AANAT gene expression trend change pattern on the time axis as a reference, and select the target genes similar to the AANAT gene expression from the entire gene.

**Figure 10:**
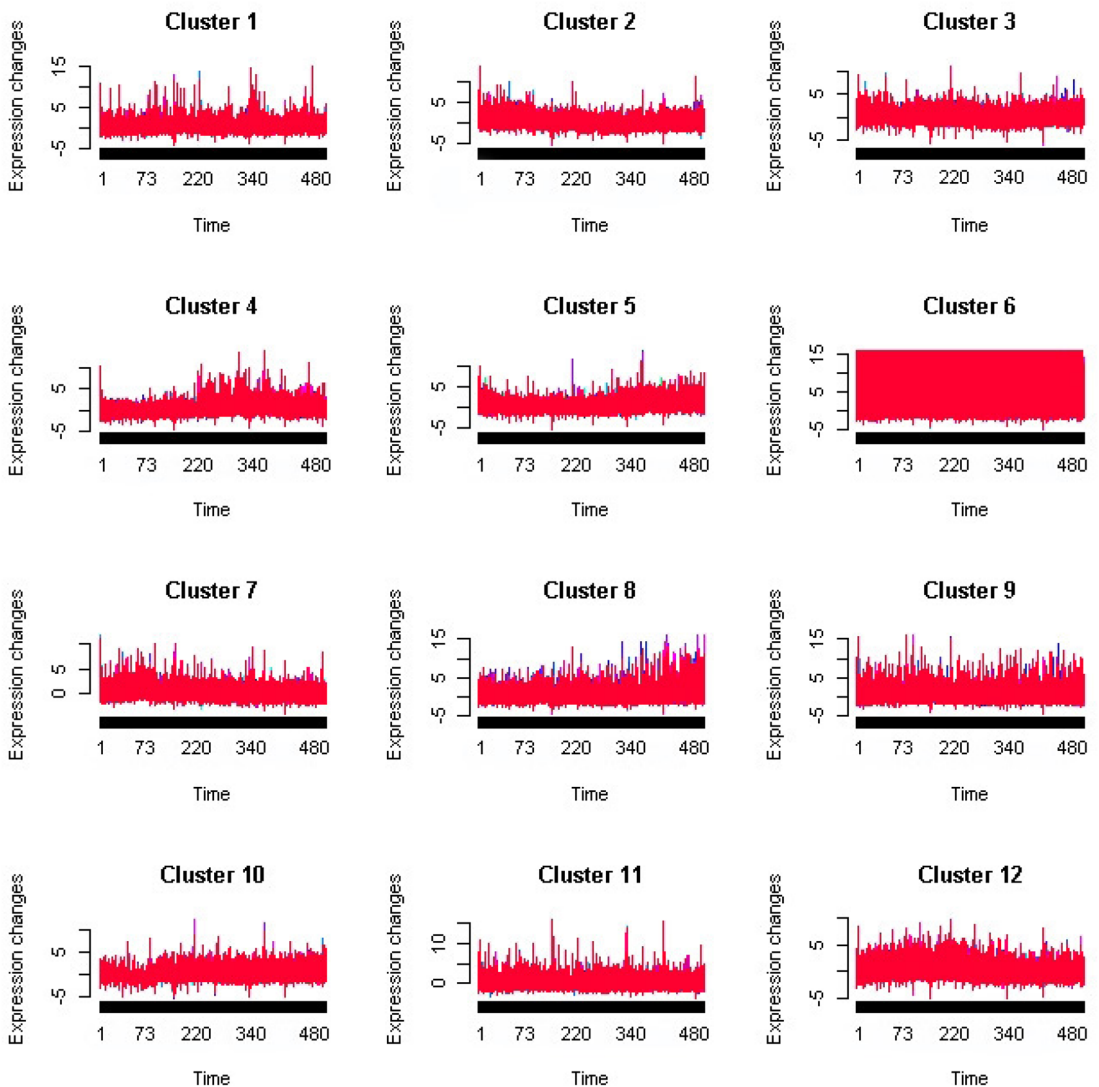
The results of cluster analysis.

We select genes with the same expression patterns of AANAT genes based on pattern clustering. At present, the popular clustering methods of time series data include fuzzy c-means method, cosine similarity method and so on. For the gene expression profile matrix of rat pineal gland, we first filter out genes whose expression levels have not changed, have no significant changes, or have a standard deviation of 0 over time. Then, the remaining genes are analyzed by pattern clustering based on the fuzzy c-means method. We divided the genes into 12 categories, and the pattern clustering results are shown in Fig. 10. Finally, we take the category of genes containing AANAT as target genes. The AANAT gene is contained in cluster 4, and the number of genes is 883.

**Figure 11:**
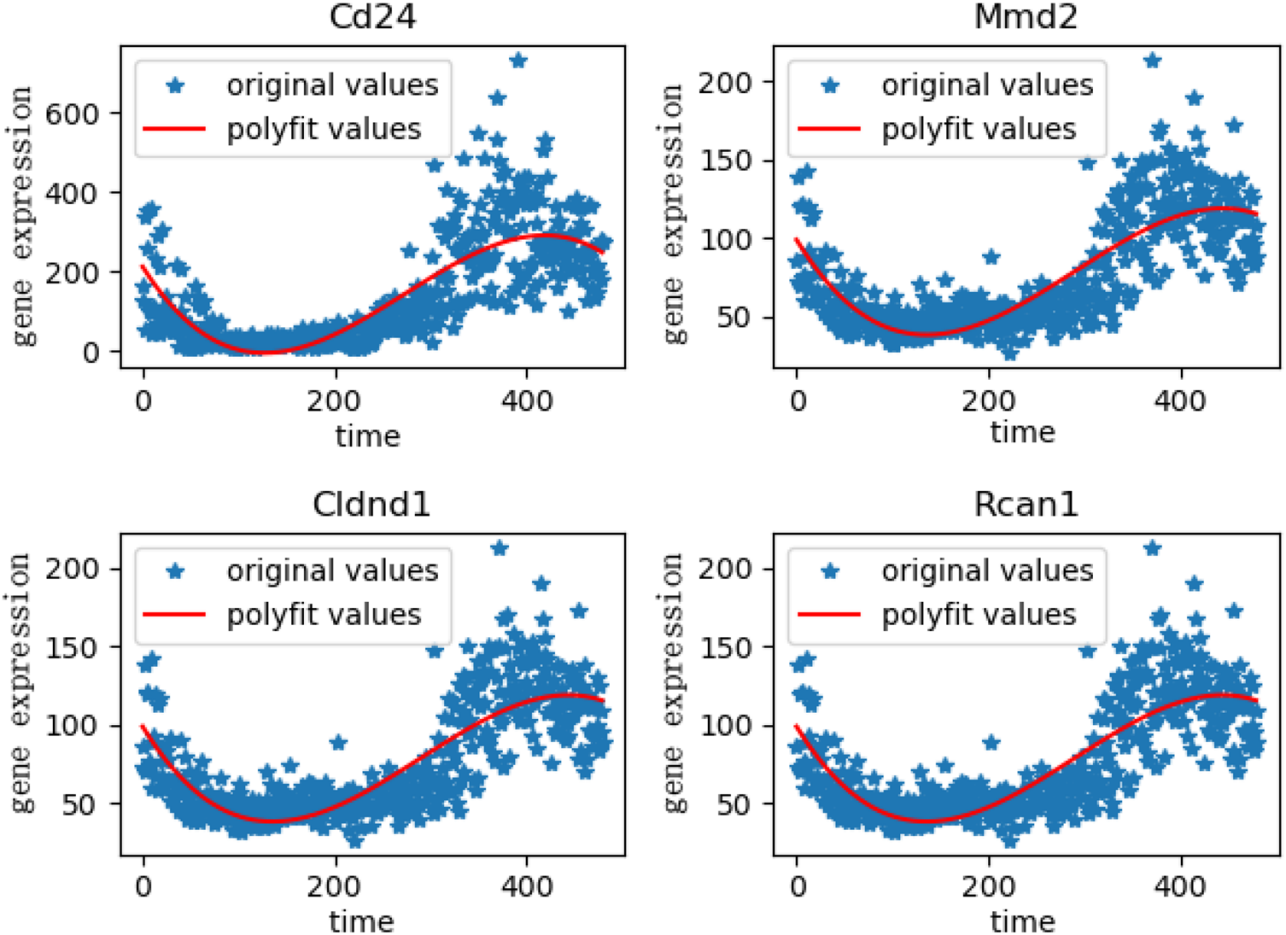
Some genes in the screening results.

In order to obtain better clustering results, we also perform cluster analysis based on the method of cosine similarity clustering. Finally, we selected the category that contains AANAT genes, which contains 643 genes. In view of the results of the two clustering methods, we selected the genes shared by the two clustering methods, and 350 target rhythm genes were obtained in this process. Then we manually screened 350 genes and removed genes that did not meet the expression trend of AANAT genes on the time axis. The change trend of target gene expression is shown in Fig. 11. Finally, 124 genes were selected to construct a gene regulatory network.

In the stage of in the stage of calculating transfer entropy, we screened out 124 genes with similar expression patterns to AANAT genes, and each gene contains 480 time points of gene expression information. These genes can be represented by an expression profile matrix in n × t format, where n represents the number of genes and t represents the length of the time sequence. Therefore, the gene expression data we collected based on time series accord with the application advantages of transferring entropy to deal with large-scale time series, and the causal relationship between gene pairs can be established by transferring entropy.

The gene expression data we collected based on time series has the characteristics of large scale, which is in line with the application advantage of transfer entropy. In addition, the causal regulation relationship between gene pairs can be established based on the transfer of entropy. Due to the asymmetry of the transfer entropy, we need to calculate the two-way transfer entropy between the pair of genes. The calculation of the total number of transfer entropy is shown in Eq. (7).

7 $$ \begin{aligned} m & = A_{n}^{2} \\ & = n \times (n - 1) \\ & = n^{2} - n \\ \end{aligned} $$

where m represents the total number of relationships, n represents the number of genes. From formula (1), we can see that the total numbers of transfer entropy that needs to be calculated increases by n^2^. Therefore, we can obtain 15,252 gene regulatory relationships based on the analysis of 124 genes. In this process, we get the values of transfer entropy and p-value between all paired genes.

**Figure 12:**
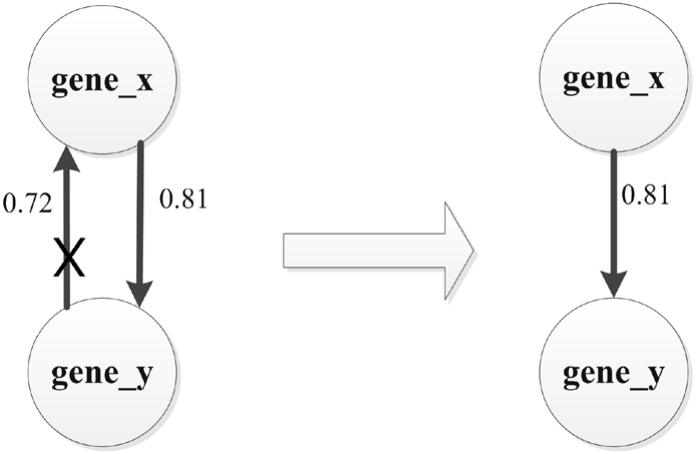
Screening for one-way gene regulatory relationships.

In the stage of in the stage of regulatory relationship screening, the screening of regulatory relationships between genes is one of the key steps in the constructing of gene regulatory networks. We screened gene regulatory relationships based on the transfer entropy and p-value between paired genes. We first screen the one-way regulatory relationship between the paired genes. As shown in Fig. 12, we retain the one-way regulatory relationship between the paired genes according to the value of transfer entropy. In this process, we have obtained 7626 regulatory relationships.

Then, we screened according to the p-value value of the evaluation index of the transfer entropy between the paired genes. The regulatory relationship between paired genes that has extremely significant information transfer changes needs to be selected by us, so we retain the regulatory relationship with p-value < 0.001. In the process, we can get 7243 gene regulation relationships with statistical significance. Finally, we further screened gene regulation relationships with TE ≥ 0.5 and obtained 743 gene regulation relationships.

**Figure 13:**
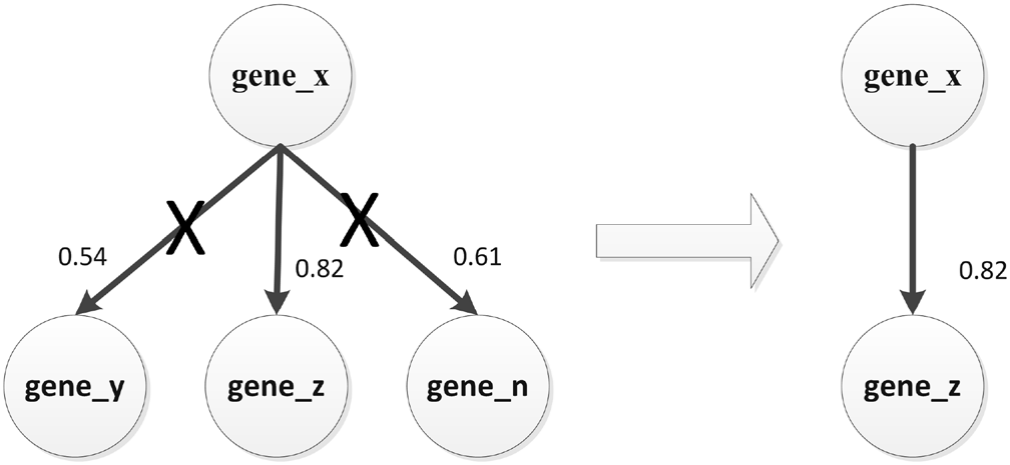
Screen the strongest gene regulatory relationship.

In the stage of gene regulation network construction, we need to screen the major gene regulation relationships. As shown in Fig. 13, we first screened the strongest gene regulatory relationship of each gene to other genes.

**Figure 14:**
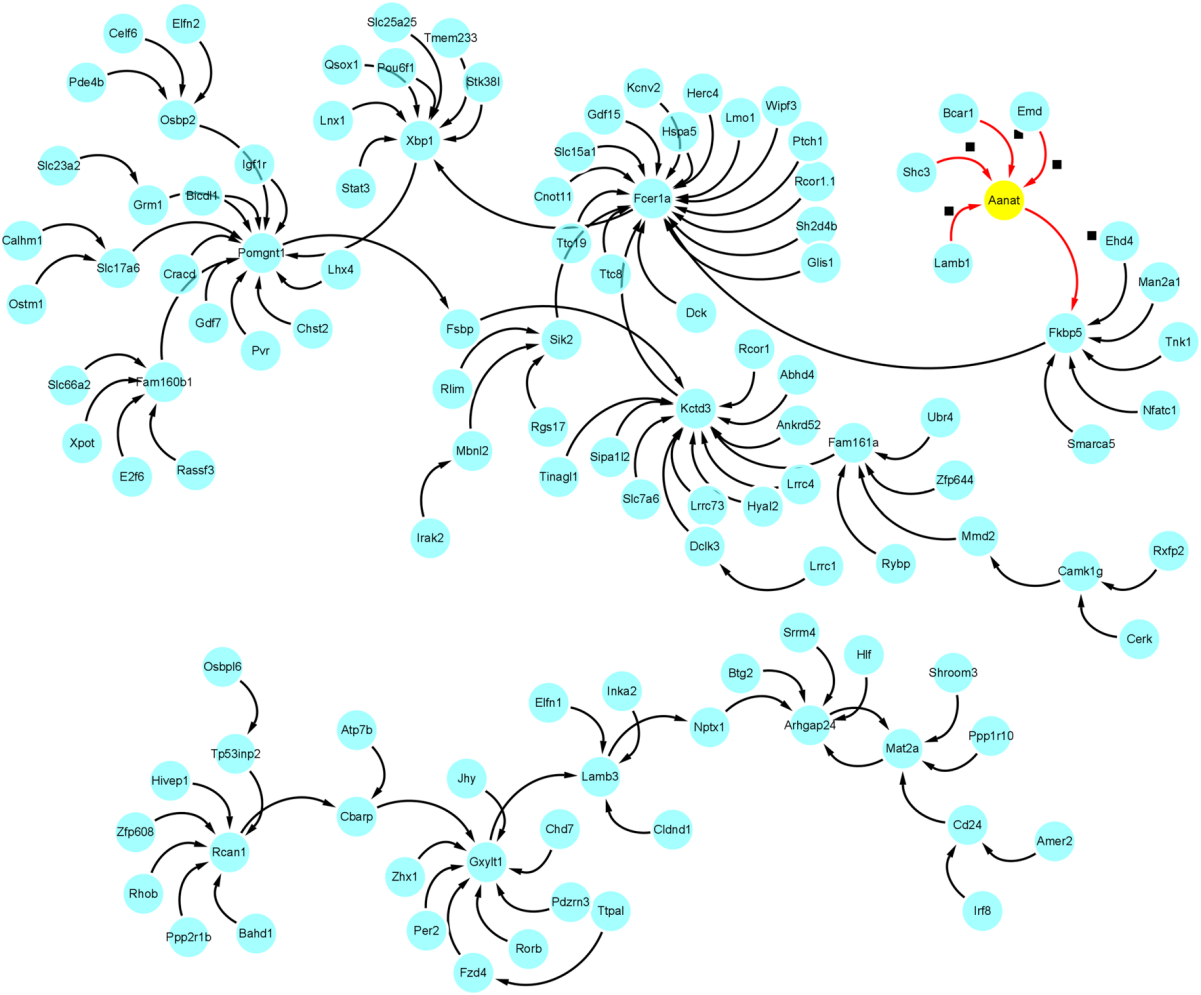
The gene regulatory network.

We finally got 117 gene regulatory relationships based on the above screening methods. Finally, the Cytoscape software was used to construct the gene regulatory network, and the gene regulatory network is shown in Fig. 14.

**Figure 15:**
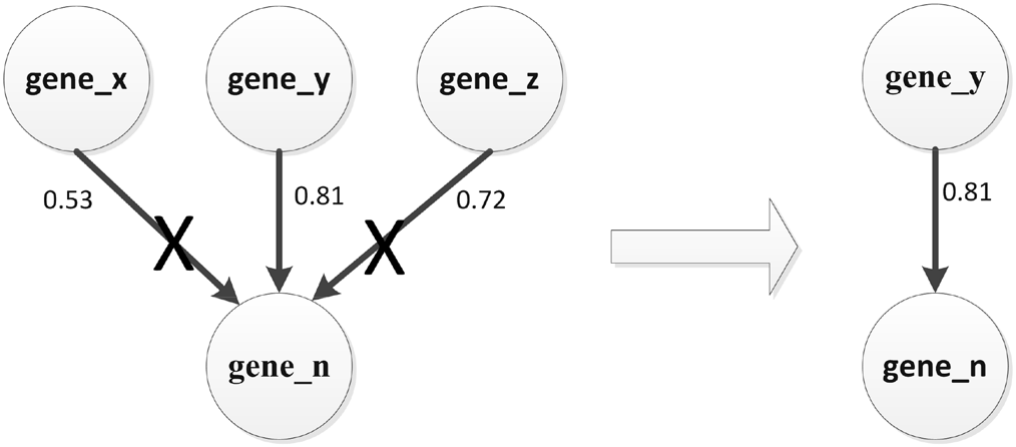
Screen the strongest gene regulatory relationship.

In addition, as shown in Fig. 15, we screened the strongest gene regulatory relationship of other genes to each gene.

**Figure 16:**
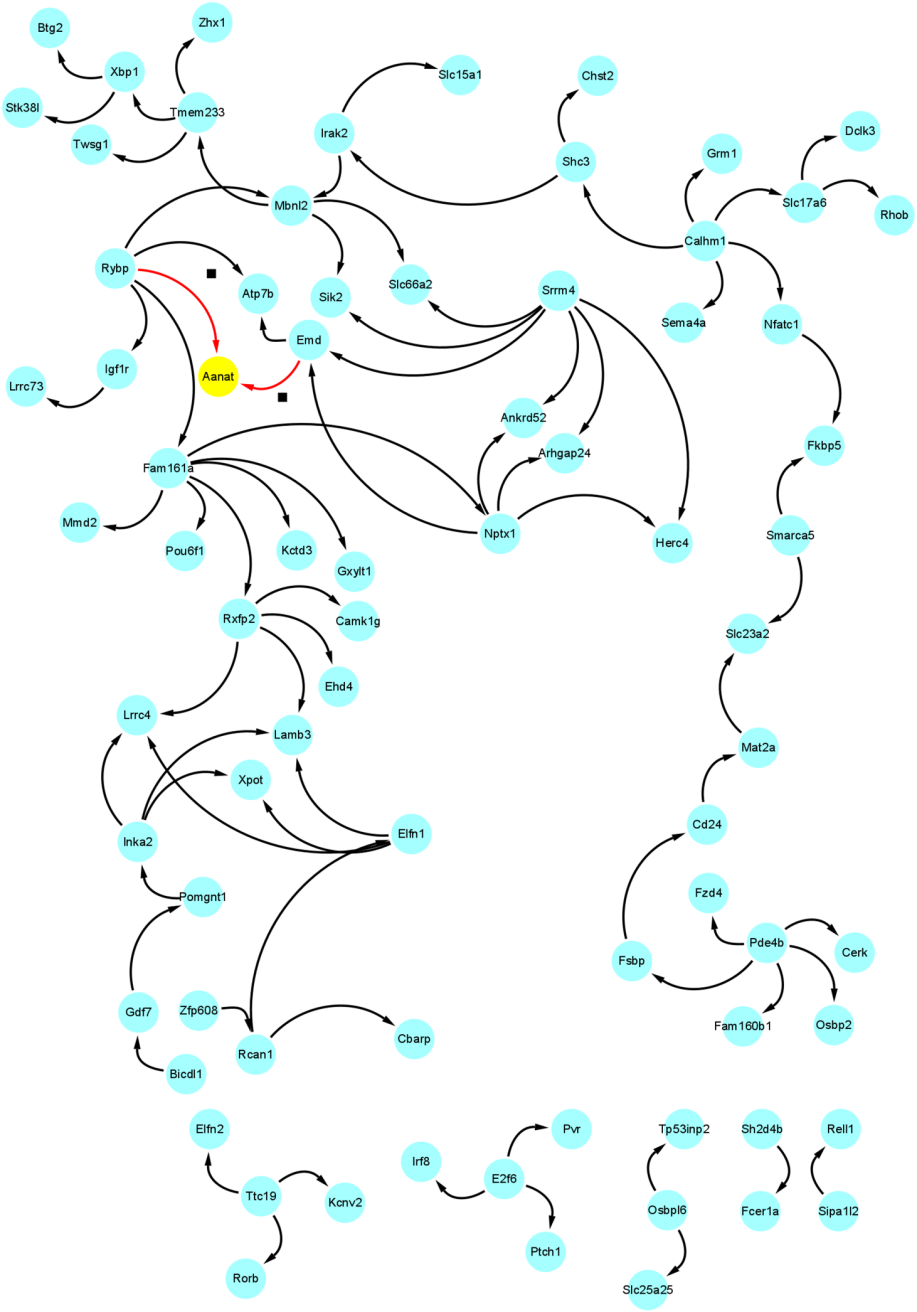
The gene regulatory network.

We finally got 80 gene regulatory relationships based on the above screening methods. Finally, the Cytoscape software was used to construct the gene regulatory network, and the gene regulatory network is shown in Fig. 16.

In summary, our experimental analysis results show that the AANAT gene is the ultimate receptor gene and is highly related to the secretion of melatonin. It is consistent with the conclusion that Binkley^57–61^ found that the daily synthesis and secretion of melatonin in the rat pineal gland is highly correlated with N-acetyltransferase activity, which also shows the effectiveness of our method. In addition, many other rhythm genes (fcer1a, XBP1, FKBP5, camk1g, RCAN1, Per2 and so on) are also included in the gene regulatory network we constructed, which have been verified by researchers through experiments such as gene knockout. For example, Wang et al.^62^ proved that the fcer1a gene is an important rhythm gene, and the expression of fcer1a gene and FceRIa protein displayed a circadian pattern following serum shock, with mean periods of 18.9 and 28.6 h, respectively. Pan et al.^63^ show that in mouse liver, transcriptional regulation significantly contributes to the establishment of 12-h rhythms of mRNA expression in a manner dependent on Spliced Form of X-box Binding Protein 1 (XBP1s). Mechanistically, the motif stringency of XBP1s promoter binding sites dictates XBP1s's ability to drive 12-h rhythms of nascent mRNA transcription at dawn and dusk. Terrelonge et al.^64^ KIBRA, MTNR1B, and FKBP5 play an important roles in the complex relationship between delirium, cognition, and sleep, and warrant further study in larger, more diverse populations. Secretion of the stress hormone cortisol follows a circadian rhythm and is stimulated following stress exposure. Yurtsever et al.^65^ studied the temporal association between unstimulated, diurnal cortisol secretion and the expression of selected GR-target genes (PER1, PER2, PER3, FKBP5, GILZ and SDPR) in vivo to determine the timing of the most pronounced coupling between cortisol and mRNA expression. Adi et al.^66^ have implicated one rhythmically expressed gene, camk1gb, in connecting the clock with downstream physiology of the pineal gland. Remarkably, knockdown of camk1gb disrupts locomotor activity in the whole larva, even though it is predominantly expressed within the pineal gland. Therefore, it appears that camk1gb plays a role in linking the pineal master clock with the periphery. Wong et al.^67^ demonstrated that both loss and aberrant gain of RCAN1precipitate anomalous light-entrained diurnal and circadian activity patterns emblematic of DS, AD, and aging by gene knockout experiments. In conclusion, the above studies not only fully proved the effectiveness of GRNTSTE method, but also proved that the gene regulatory network we constructed has important reference value.

## Discussion

In our work, in order to infer the gene regulation relationship from the massive time series gene expression data, we propose the GRNTSTE method, which uses transfer entropy to infer the regulatory relationship between genes. We compared GRNTSTE with the existing algorithms SCRBE, TENET and dynGENIE3, and the results show that GRNTSTE has better performance than dynGENIE3 and SCRBE. However, GRNTSTE and TENET have similar performance. At the same time, we prove the performance of GRNTSTE is slightly lower than that of the SINCERITIES method, and it outperforms other gene regulatory network construction methods in BEELINE. It shows the superiority of GRNTSTE in reconstructing gene regulatory networks based on single-cell gene expression data. Then, we applied the GRNTSTE method to the construction of the rhythm gene regulatory network in rat pineal gland tissue. The gene regulatory network constructed based on large-scale time series gene expression data is helpful for studying the interaction between rhythm genes. It is great significance to explore the interaction between genes that secrete melatonin in the pineal gland. It is great significance to comprehensively explain the molecular mechanism of melatonin secretion. In addition, it can guide and treat diseases caused by the pineal gland, such as insomnia.

Aromatic alkylamine N-acetyltransferase in the pineal gland is an important rate-limiting enzyme in the melatonin biosynthesis pathway. It may be involved in regulating the synthesis rhythm of melatonin, and it may play an important role in influencing the regulation of the photoperiod to the night peak of melatonin. In the pineal gland of normal rats, the AANAT is a soluble cytoplasmic protein. The enzyme activity of AANAT is high at night and low during the day. In addition, light can quickly reduce the AANAT enzyme activity, and compared with the activities of other enzymes in the process of melatonin synthesis, the AANAT activity is extremely low during the day. It shows that AANAT is the main rate-limiting enzyme in the process of melatonin synthesis. The periodic changes of AANAT activity in the pineal gland of most mammals can drive the circadian secretion of melatonin. Therefore, AANAT is called the melatonin rhythm-forming enzyme.

In order to study the regulatory relationship between rhythm genes in rat pineal tissue, we adopted the controlled variable method. The sampling interval is 3 min, and the sampling time is 24 h. We obtained 480 rat pineal tissue samples to form a time series gene sample data set. Large-scale time series data serve as our basic data set for constructing gene regulatory networks. The method replaces the traditional two-state or a small amount of time points data with large-scale time series data. We break through the traditional genetic data analysis model and propose a new analysis method GRNTSTE for the study of dynamic biological processes.

Then, we choose the rate-limiting enzyme AANAT for melatonin synthesis as the starting point of the research object. We obtained the rhythm target genes similar to the expression pattern of the AANAT gene on the time axis based on the clustering method. And we construct a gene regulatory network of rhythm genes in rat pineal tissue based on large-scale gene representation time series data and transfer entropy, in which the transfer entropy is used to infer the gene regulatory relationship. And our experimental results are highly consistent with existing research, which provides a very valuable reference basis for further biological experiment verification.

The GRNTSTE method breaks through the traditional way of gene regulatory network construction, and it is the first time to explore the regulatory network relationship between genes based on a data-driven model. And the construction of gene regulatory network by GRNTSTE method is based on large-scale data-driven analysis of genomics data, which effectively avoids the misleading caused by the randomness of gene expression data. In addition, large-scale time series data can effectively reflect the dynamic biological process information of gene expression levels. Therefore, the GRNTSTE method can not only effectively construct a gene expression regulatory network and provide a valuable basis for the in-depth exploration of biological experiments, but also can effectively avoid the huge cost waste caused by blind biological experiments. The method proposed in this paper provides a new analysis idea for the study of gene regulatory network, which has theoretical and practical value.

## Conclusions

The systems biology method of constructing gene regulatory network based on large-scale time series data can provide reference basis and hypothesis for biological experiment verification. However, there are few methods to construct gene expression regulatory networks based on large-scale time-series gene expression data, and existing methods cannot well capture continuous cell dynamics and dynamic biological processes.

In this paper, we first collected the time series data of the rat pineal gland tissue in the natural state according to a fixed sampling rate, and performed whole-genome sequencing. The large-scale time-series sequencing data set of rat pineal gland was constructed, which includes 480 time points, the time interval between adjacent time points is 3 min, and the sampling period is 24 h.

Then, we proposes a method named GRNTSTE for constructing gene regulatory networks based on large-scale time series data. We prove that the GRNTSTE algorithm has better performance than SCRIBE and dynGENIE3 based on the DREAM3 challenge data set. However, GRNTSTE and TENET have similar performance. At the same time, we compare and analyze the gene regulatory network method in the BEELINE framework and GRNTSTE based on the BEELINE single-cell datasets. It proves that the performance of GRNTSTE is slightly lower than that of SINCERITIES method and better than other gene regulatory network construction methods in BEELINE framework, which is based on the BEELINE data set. It shows the effectiveness and superiority of GRNTSTE in reconstructing gene regulatory networks based on single-cell gene expression data. In addition, we further verify the effectiveness of our proposed gene regulatory network inference method GRNTSTE based on public datasets named IRMA OFF/ON from Cantone et al. Comparing ODE-Based, the GRNTSTE has higher sensitivity when PPV is similar.

Finally, take the rhythm gene in the pineal gland of the rat as an example, the transfer entropy is used to evaluate the regulatory relationship between gene pairs, and the rat pineal rhythm gene regulatory network is constructed based on the GRNTSTE algorithm. And in the gene regulatory network we constructed, many genes are consistent with the existing research results. It provides a valuable reference for the study of the regulation mechanism of pineal rhythm. It is of great significance to the study of dynamic biological processes.

## Data Availability

Our datasets has been uploaded to the NCBI public database. And we are also working on the database of rat rhythm center, so we will publish it later. SRA number of sequencing data: SRR18934928–SRR18935407.
